# Supplementary material for: Associations Between Hemoglobin and Serum Iron Levels and the Risk of Mortality Among Patients with Coronary Artery Disease
Source: Nutrients. 2024 Dec 31;17(1):139. doi: 10.3390/nu17010139 (PMC11722639; doi:10.3390/nu17010139)
Supplement: Supplementary file 1 [file nutrients-17-00139-s001.zip › nutrients-3382366-supplementary.pdf]

Supplementary Table S1. Correlations of serum iron and hemoglobin levels with cardiology risk factors.

Variables	Serum iron		Hemoglobin	
	No Adjustment†	Adjusted by sex, age, body mass index ‡	No Adjustment†	Adjusted by sex, age, body mass index ‡
**Serum iron**	--	--	0.295**	0.259**
**Hemoglobin**	0.295**	0.259**	--	--
**Body mass index, kg/m^2**	0.059**	--	0.159**	--
**Sex**	-0.059**	--	-0.385**	--
**Age**	-0.096**	--	-0.309**	--
**Systolic blood pressure, mmHg**	0.037*	0.038*	-0.005	0.069**
**Total cholesterol, mmol/L**	0.090**	0.079**	0.091**	0.147**
**Low-density lipoprotein cholesterol, mmol/L**	0.070**	0.051**	0.101**	0.119**
**High-density lipoprotein cholesterol, mmol/L**	0.159**	0.183**	0.021	0.137**

**Triglycerides, mmol/L**	0.054**	-0.011	0.151**	0.072**
**C-reactive protein#, mg/L**	-0.398**	-0.114**	-0.403**	-0.140**
**Fasting plasma glucose, mmol/L**	-0.094**	-0.065**	-0.040*	-0.052**

** Correlation is significant at the 0.01 level (2-tailed).

* Correlation is significant at the 0.05 level (2-tailed).

† Spearman correlation analysis was used.

‡ Partial correlation analysis was used.

# Log transformed before analysis.

Supplementary Table S2. Hazard ratios for cardiovascular and all-cause mortality according to hemoglobin levels among various subgroups.

	Cardiovascular mortality			All-cause mortality		
	**Hazard Ratio per 1-SD Increment**	***P* value**	***P* for interaction**	**Hazard Ratio per 1-SD Increment**	***P* value**	***P* for interaction**
Age			0.903			0.117
≥ 65 (n = 1,611)	0.78 (0.68–0.88)	<0.001		0.76 (0.68–0.83)	<0.001	
< 65 (n = 1,613)	0.73 (0.60–0.89)	0.002		0.81 (0.69–0.96)	0.016	
Type of CAD			0.289			0.014
Acute coronary artery disease (*n* = 1,823)	0.82 (0.72–0.95)	0.007		0.77 (0.692–0.86)	<0.001	
Stable coronary artery disease (*n* = 1,401)	0.78 (0.66–0.92)	0.004		0.85 (0.75–0.98)	0.022	
Sex			0.058			0.104

Male (n = 2,052)	0.84 (0.74–0.96)	0.009		0.83 (0.75–0.92)	<0.001	
Female (n = 1,172)	0.74 (0.59–0.92)	0.006		0.76 (0.65–0.90)	0.001	
Body mass index, kg/m^2			0.740		0.106	
≥ 24 (n = 1,537)	0.83 (0.71–0.99)	0.034		0.86 (0.75–0.98)	0.028	
< 24 (n = 1,687)	0.80 (0.69–0.92)	0.002		0.79 (0.70–0.88)	<0.001	
History of diabetes			0.004			0.001
No (n = 2,461)	0.84 (0.74–0.96)	0.013		0.82 (0.74–0.91)	<0.001	
Yes (n = 763)	0.74 (0.61–0.90)	0.002		0.78 (0.68–0.91)	0.001	

Adjusted for age, sex, drinking alcohol status, smoking status, body mass index, systolic blood pressure, diastolic blood pressure, fasting plasma glucose, non-high-density lipoprotein cholesterol, triglycerides, duration of coronary artery disease, estimated glomerular filtration rate, type of coronary artery disease (acute and chronic), C-reactive protein, use of anti-diabetic, anti-platelet, cholesterol-lowering and anti-hypertensive drugs, and serum iron levels.

Supplementary Table S3. Hazard ratios for cardiovascular and all–cause mortality according to serun iron levels among various subgroups.

	**Cardiovascular mortality**			**All–cause mortality**		
	**Hazard Ratio per 1–SD Increment**	***P* value**	***P* for interaction**	**Hazard Ratio per 1–SD Increment**	***P* value**	***P* for interaction**
Age			0.116			0.760
≥ 65 (n = 1,611)	0.77 (0.66–0.88)	<0.001		0.82 (0.74–0.91)	<0.001	
< 65 (n = 1,613)	0.78 (0.62–0.97)	0.025		0.83 (0.69–0.99)	0.044	
Type of CAD			0.408			0.075
Acute coronary artery disease (*n* = 1,823)	0.78 (0.66–0.91)	0.002		0.79 (0.69–0.89)	<0.001	
Stable coronary artery disease (*n* = 1,401)	0.80 (0.66–0.97)	0.024		0.89 (0.76–1.03)	0.117	
Sex			0.500			0.763

Male (*n* = 2,052)	0.80 (0.69–0.92)	0.003		0.84 (0.75–0.94)	0.003	
Female (*n* = 1,172)	0.72 (0.58–0.90)	0.003		0.78 (0.66–0.92)	0.004	
Body mass index, kg/m^2			0.899			0.191
≥ 24 (*n* = 1,537)	0.76 (0.63–0.92)	0.004		0.81 (0.70–0.94)	0.007	
< 24 (*n* = 1,687)	0.81 (0.69–0.95)	0.011		0.86 (0.76–0.97)	0.016	
History of diabetes			0.056			0.034
No (*n* = 2,461)	0.86 (0.75–1.00)	0.047		0.89 (0.80–1.00)	0.050	
Yes (*n* = 763)	0.63 (0.50–0.79)	<0.001		0.71 (0.59–0.84)	<0.001	

Adjusted for age, sex, drinking alcohol status, smoking status, body mass index, systolic blood pressure, diastolic blood pressure, fasting plasma glucose, non-high-density lipoprotein cholesterol, triglycerides, duration of coronary artery disease, estimated glomerular filtration rate, type of coronary artery disease (acute and chronic), C-reactive protein, use of anti-diabetic, anti-platelet, cholesterol-lowering and anti-hypertensive drugs, and hemoglobin levels.

Supplementary Table S4. Hazard ratios of cardiovascular and all-cause mortality according to plasma ferritin levels.

	Plasma ferritin (n=1190)						
	Quartile 1 (n=297)	Quartile 2 (n=297)	Quartile 3 (n=299)	Quartile 4 (n=297)	*P* value	Hazard Ratio per 1-SD Increment [#]	*P* value
**Median [25th, 75th], ng/mL**	111.3 (76.4–148.1)	227.2 (196.4–281.4)	357.5 (304.5–413.8)	629.8 (514.8–869.2)	<0.001	-	-
**Cardiovascular mortality**							
Model 1	1.00	1.30 (0.89–1.92)	1.01 (0.66–1.55)	1.64 (1.12–2.38)	0.010	1.26 (1.08–1.47)	0.003
Model 2	1.00	1.37 (0.92–2.03)	1.08 (0.70–1.66)	1.52 (1.02–2.25)	0.132	1.22 (1.05–1.43)	0.012
Model 3	1.00	1.40 (0.94–2.08)	1.15 (0.74–1.78)	1.55 (1.04–2.31)	0.132	1.25 (1.08–1.45)	0.003
**All-cause mortality**							
Model 1	1.00	1.10 (0.80–1.52)	1.12 (0.80–1.55)	1.48 (1.09–2.01)	0.013	1.19 (1.06–1.35)	0.005

Model 2	1.00	1.16 (0.83–1.60)	1.19 (0.85–1.67)	1.42 (1.03–1.96)	0.207	1.17 (1.04–1.33)	0.012
Model 3	1.00	1.19 (0.85–1.65)	1.25 (0.89–1.76)	1.45 (1.05–2.01)	0.163	1.21 (1.07–1.36)	0.002

Model 1 was adjusted for age, sex, drinking alcohol status, and smoking status.

Model 2 was adjusted for model 1 covariates plus body mass index, systolic blood pressure, diastolic blood pressure, fasting plasma glucose, non-high-density lipoprotein cholesterol, triglycerides, duration of coronary artery disease, estimated glomerular filtration rate, type of coronary artery disease (acute and chronic), C-reactive protein, use of anti-diabetic, anti-platelet, cholesterol-lowering and anti-hypertensive drugs.

Model 3 was adjusted for model 2 covariates plus serum iron and hemoglobin levels.

[#] Log transformed before analysis.

Supplementary Table S5. Hazard ratios of cardiovascular and all-cause mortality according to serum transferrin levels.

	**Serum transferrin (n=1473)**						
	Quartile 1 (n=366)	Quartile 2 (n=373)	Quartile 3 (n=361)	Quartile 4 (n=373)	*P* value	Hazard Ratio per 1-SD Increment	*P* value
**Median [25th, 75th], g/L**	1.71 (1.58–1.80)	2.02 (1.95–2.07)	2.26 (2.20–2.33)	2.60 (2.48–2.82)	<0.001	-	-
**Cardiovascular mortality**							
Model 1	1.00	0.74 (0.48–1.15)	0.46 (0.27–0.77)	0.63 (0.38–1.03)	0.022	0.74 (0.61–0.90)	0.003
Model 2	1.00	0.74 (0.48–1.15)	0.45 (0.27–0.76)	0.62 (0.37–1.02)	0.020	0.73 (0.60–0.90)	0.002
Model 3	1.00	0.85 (0.55–1.32)	0.51 (0.30–0.86)	0.77 (0.45–1.29)	0.093	0.81 (0.66–0.99)	0.038
**All-cause mortality**							
Model 1	1.00	0.76 (0.54–1.08)	0.59 (0.40–0.87)	0.81 (0.56–1.18)	0.063	0.88 (0.76–1.02)	0.092

Model 2	1.00	0.75 (0.53–1.07)	0.58 (0.39–0.86)	0.83 (0.57–1.23)	0.050	0.89 (0.76–1.03)	0.130
Model 3	1.00	0.86 (0.60–1.23)	0.66 (0.44–0.98)	1.05 (0.71–1.57)	0.118	0.98 (0.84–1.14)	0.764

Model 1 was adjusted for age, sex, drinking alcohol status, and smoking status.

Model 2 was adjusted for model 1 covariates plus body mass index, systolic blood pressure, diastolic blood pressure, fasting plasma glucose, non-high-density lipoprotein cholesterol, triglycerides, duration of coronary artery disease, estimated glomerular filtration rate, type of coronary artery disease (acute and chronic), C-reactive protein, use of anti-diabetic, anti-platelet, cholesterol-lowering and anti-hypertensive drugs.

Model 3 was adjusted for model 2 covariates plus serum iron and hemoglobin levels.

Supplementary Table S6. Hazard ratios of cardiovascular and all-cause mortality according to total iron binding capacity.

	**Total iron binding capacity (n=1470)**						
	Quartile 1 (n=366)	Quartile 2 (n=365)	Quartile 3 (n=371)	Quartile 4 (n=368)	*P* value	Hazard Ratio per 1-SD Increment	*P* value
**Median [25th, 75th], µmol/L**	36.7 (34.0–38.6)	42.7 (41.3–43.9)	47.0 (46.0–48.3)	53.8 (51.1–57.7)	<0.001	-	-
**Cardiovascular mortality**							
Model 1	1.00	0.84 (0.55–1.29)	0.51 (0.32–0.86)	0.51 (0.29–0.87)	0.017	0.74 (0.62–0.89)	0.002
Model 2	1.00	0.82 (0.53–1.27)	0.50 (0.30–0.84)	0.48 (0.28–0.84)	0.014	0.72 (0.60–0.88)	0.001
Model 3	1.00	0.91 (0.59–1.42)	0.59 (0.35–0.99)	0.60 (0.34–1.08)	0.125	0.81 (0.66–0.99)	0.037
**All-cause mortality**							
Model 1	1.00	0.78 (0.55–1.09)	0.58 (0.39–0.84)	0.70 (0.47–1.04)	0.035	0.85 (0.74–0.98)	0.029

Model 2	1.00	0.79 (0.55–1.11)	0.57 (0.38–0.84)	0.70 (0.47–1.04)	0.038	0.85 (0.73–0.99)	0.038
Model 3	1.00	0.89 (0.62–1.27)	0.66 (0.45–0.99)	0.91 (0.60–1.39)	0.239	0.96 (0.82–1.12)	0.592

Model 1 was adjusted for age, sex, drinking alcohol status, and smoking status.

Model 2 was adjusted for model 1 covariates plus body mass index, systolic blood pressure, diastolic blood pressure, fasting plasma glucose, non-high-density lipoprotein cholesterol, triglycerides, duration of coronary artery disease, estimated glomerular filtration rate, type of coronary artery disease (acute and chronic), C-reactive protein, use of anti-diabetic, anti-platelet, cholesterol-lowering and anti-hypertensive drugs.

Model 3 was adjusted for model 2 covariates plus serum iron and hemoglobin levels.

Supplementary Table S7. Hazard ratios of cardiovascular and all-cause mortality according to transferrin saturation.

	Transferrin saturation (n=1470)						
	Quartile 1 (n=367)	Quartile 2 (n=368)	Quartile 3 (n=368)	Quartile 4 (n=367)	*P* value	Hazard Ratio per 1-SD Increment	*P* value
**Median [25th, 75th], %**	17.6 (14.1–20.8)	27.3 (25.4–29.6)	35.5 (33.5–38.3)	47.5(43.2–54.3)	<0.001	-	-
**Cardiovascular mortality**							
Model 1	1.00	0.69 (0.45–1.08)	0.41 (0.24–0.69)	0.57 (0.36–0.91)	0.005	0.79 (0.65–0.95)	0.015
Model 2	1.00	0.64 (0.41–0.99)	0.41 (0.24–0.69)	0.54 (0.33–0.86)	0.003	0.78 (0.64–0.95)	0.016
Model 3	1.00	0.67 (0.42–1.04)	0.45 (0.26–0.76)	0.60 (0.37–0.98)	0.017	0.83 (0.68–1.00)	0.049
**All-cause mortality**							
Model 1	1.00	0.72 (0.51–1.00)	0.48 (0.33–0.70)	0.47 (0.32–0.69)	<0.001	0.73 (0.63–0.85)	<0.001

Model 2	1.00	0.71 (0.50–1.00)	0.50 (0.34–0.74)	0.47 (0.32–0.69)	<0.001	0.74 (0.64–0.87)	<0.001
Model 3	1.00	0.74 (0.53–1.05)	0.55 (0.37–0.81)	0.52 (0.35–0.78)	0.003	0.79 (0.67–0.92)	0.002

Model 1 was adjusted for age, sex, drinking alcohol status, and smoking status.

Model 2 was adjusted for model 1 covariates plus body mass index, systolic blood pressure, diastolic blood pressure, fasting plasma glucose, non-high-density lipoprotein cholesterol, triglycerides, duration of coronary artery disease, estimated glomerular filtration rate, type of coronary artery disease (acute and chronic), C-reactive protein, use of anti-diabetic, anti-platelet, cholesterol-lowering and anti-hypertensive drugs.

Model 3 was adjusted for model 2 covariates plus hemoglobin levels.

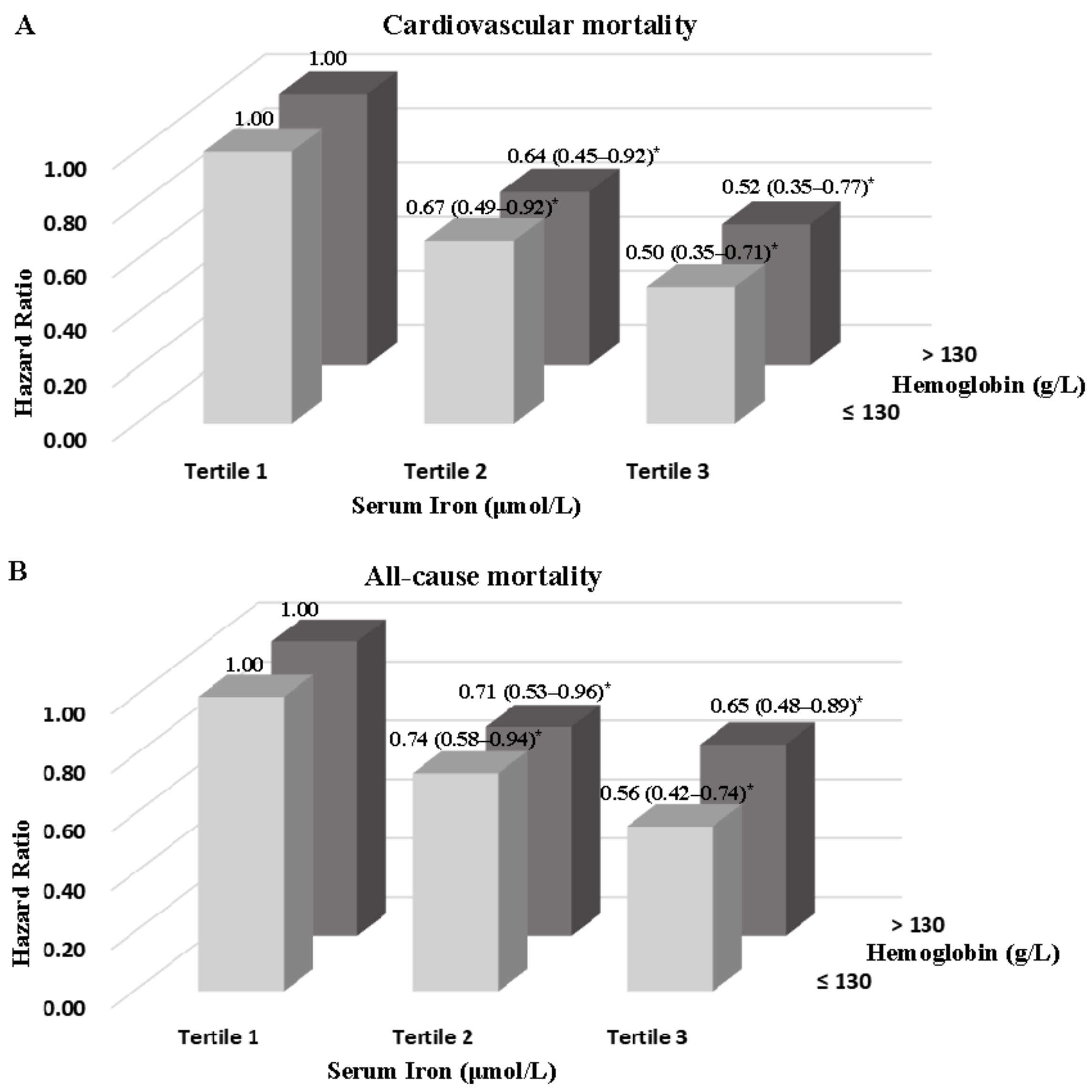

Supplementary Figure S1. Multivariable adjusted hazard ratios for cardiovascular (A) and all-cause (B) mortality according to different levels of hemoglobin and serum iron levels.

Adjusted for age, sex, drinking alcohol status, and smoking status, body mass index, systolic blood pressure, diastolic blood pressure, fasting plasma glucose, non-high-density lipoprotein cholesterol, triglycerides, duration of coronary artery disease, estimated glomerular filtration rate, type of coronary artery disease (acute and chronic), C-reactive protein, use of anti-diabetic, anti-platelet, cholesterol-lowering and anti-hypertensive drugs.

* $p<0.05$.

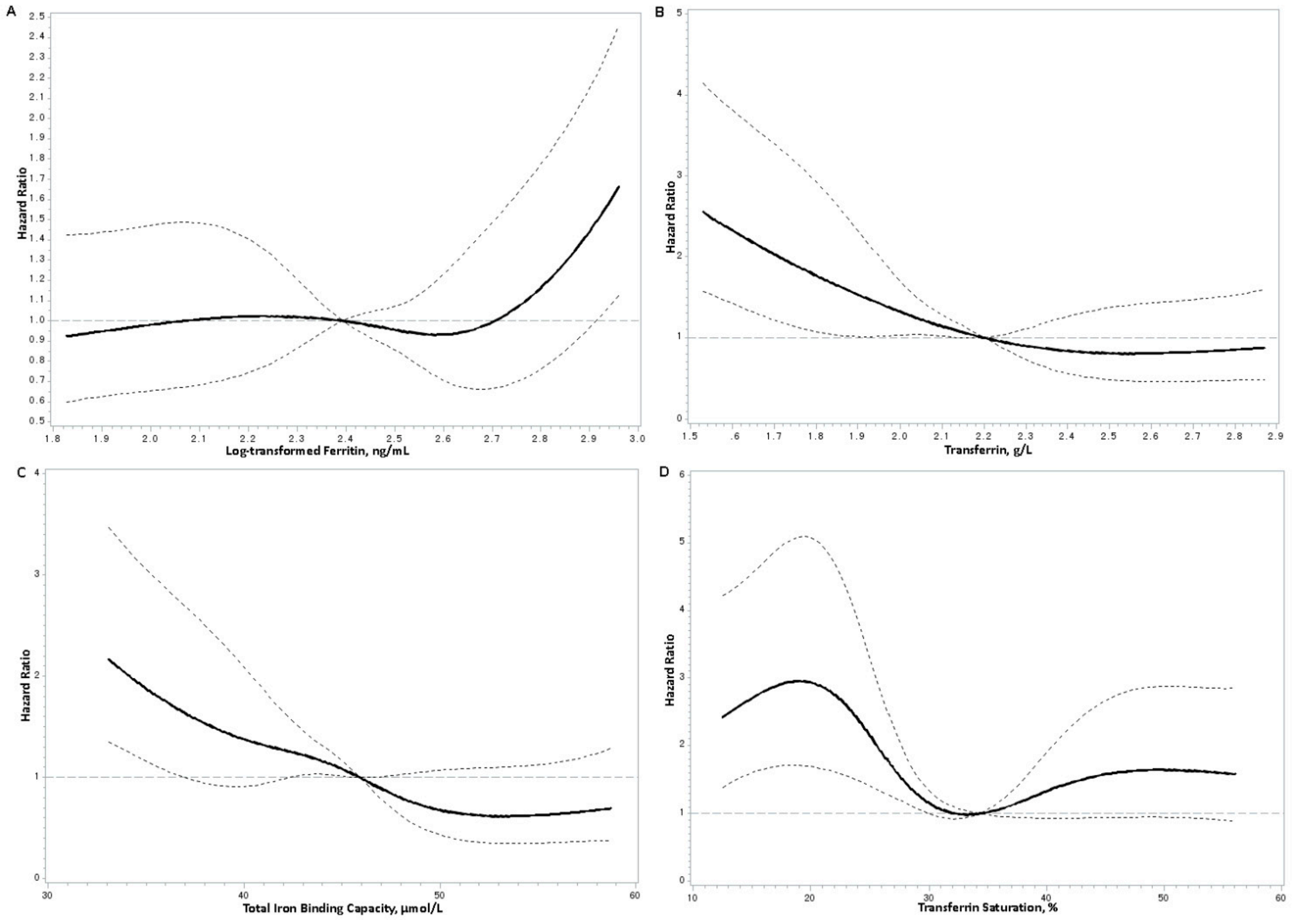

Supplementary Figure S2. Restricted cubic splines in Cox models of Log-transform ferritin (A), transferrin (B), total iron-binding capacity (C), transferrin saturation (D) levels with cardiovascular mortality risk. Adjustments were made for age, sex, drinking alcohol status, smoking status, body mass index, systolic blood pressure, diastolic blood pressure, fasting plasma glucose, non-high-density lipoprotein cholesterol, triglycerides, duration of coronary artery disease, estimated glomerular filtration rate, type of coronary artery disease (acute and chronic), C-reactive protein, use of anti-diabetic, anti-platelet, cholesterol-lowering, and anti-hypertensive drugs, hemoglobin and serum iron (except for transferrin saturation) levels.

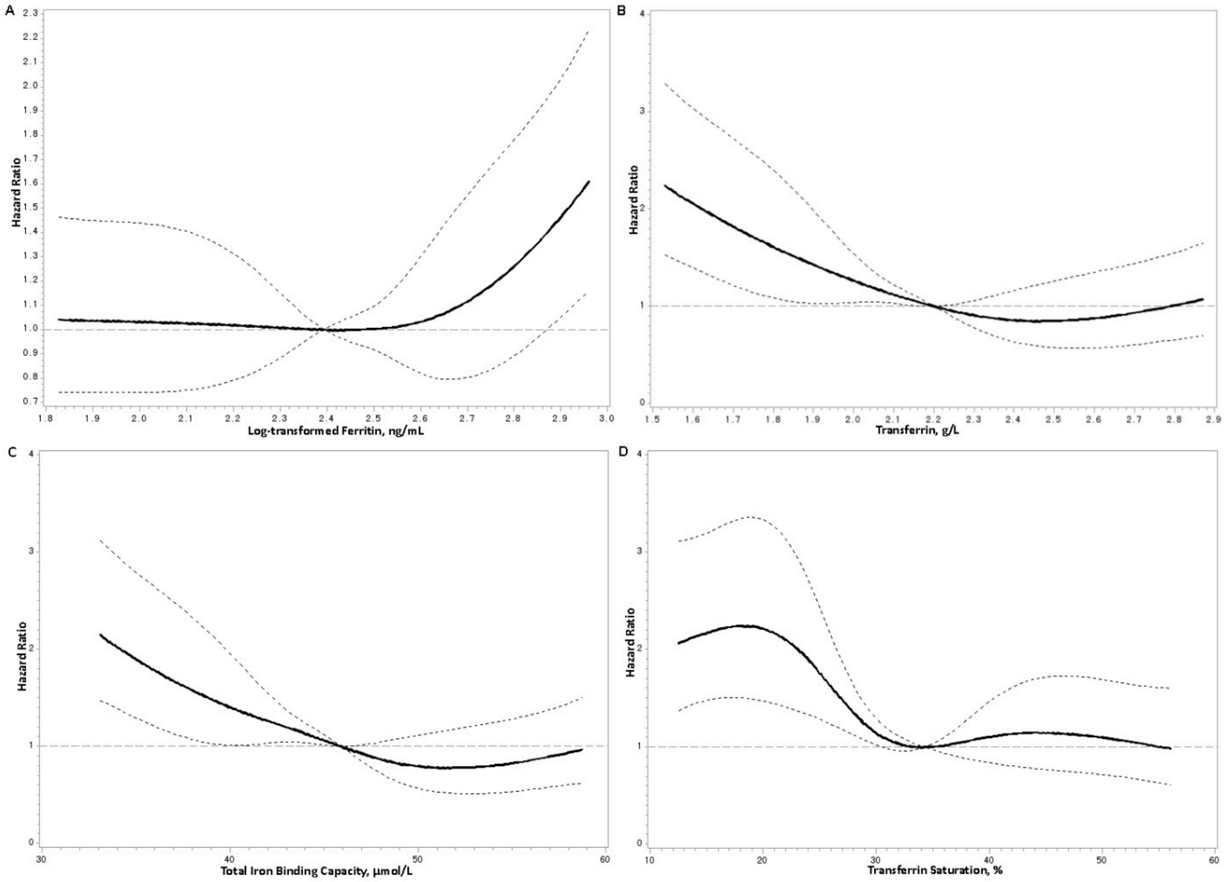

Supplementary Figure S3. Restricted cubic splines in Cox models of Log-transform ferritin (A), transferrin (B), total iron-binding capacity (C), transferrin saturation (D) levels with all-cause mortality risk. Adjustments were made for age, sex, drinking alcohol status, smoking status, body mass index, systolic blood pressure, diastolic blood pressure, fasting plasma glucose, non-high-density lipoprotein cholesterol, triglycerides, duration of coronary artery disease, estimated glomerular filtration rate, type of coronary artery disease (acute and chronic), C-reactive protein, use of anti-diabetic, anti-platelet, cholesterol-lowering, and anti-hypertensive drugs, hemoglobin and serum iron (except for transferrin saturation) levels.
